# The altered expression of perineuronal net elements during neural differentiation

**DOI:** 10.1186/s11658-018-0073-5

**Published:** 2018-02-13

**Authors:** Nazli F. Eskici, Sevim Erdem-Ozdamar, Didem Dayangac-Erden

**Affiliations:** 1 Faculty of Medicine Department of Medical Biology, Hacettepe University, Ankara, Turkey; 2 Faculty of Medicine Department of Neurology, Hacettepe University, Ankara, Turkey

**Keywords:** Perineuronal nets, HAPLN1, Tenascin-R, Aggrecan, PC12 differentiation

## Abstract

**Background:**

Perineuronal nets (PNNs), which are localized around neurons during development, are specialized forms of neural extracellular matrix with neuroprotective and plasticity-regulating roles. Hyaluronan and proteoglycan link protein 1 (HAPLN1), tenascin-R (TNR) and aggrecan (ACAN) are key elements of PNNs. In diseases characterized by neuritogenesis defects, the expression of these proteins is known to be downregulated, suggesting that PNNs may have a role in neural differentiation.

**Methods:**

In this study, the mRNA and protein levels of HAPLN1, TNR and ACAN were determined and compared at specific time points of neural differentiation. We used PC12 cells as the in vitro model because they reflect this developmental process.

**Results:**

On day 7, the HAPLN1 mRNA level showed a 2.9-fold increase compared to the non-differentiated state. However, the cellular HAPLN1 protein level showed a decrease, indicating that the protein may have roles in neural differentiation, and may be secreted during the early period of differentiation. By contrast, TNR mRNA and protein levels remained unchanged, and the amount of cellular ACAN protein showed a 3.7-fold increase at day 7. These results suggest that ACAN may be secreted after day 7, possibly due to its large amount of post-translational modifications.

**Conclusions:**

Our results provide preliminary data on the expression of PNN elements during neural differentiation. Further investigations will be performed on the role of these elements in neurological disease models.

## Background

Perineuronal nets (PNNs) are specialized substructures of the neural extracellular matrix (ECM), which surround cell soma and proximal neurites of neurons in the hippocampus, cerebellum, brain stem and spinal cord [1, 2]. PNNs are embedded in a basal lamina that includes collagen IV, laminin, heparan sulfate proteoglycans (HSPGs) and other glycoproteins [3, 4]. The major components are hyaluronan (HA), chondroitin sulfate proteoglycans (CSPGs), tenascin-R (TNR), and hyaluronan and proteoglycan link protein (HAPLN1) [5–7].

CSPGs interact with HA polymer chains on the cell surface. This interaction is stabilized by link proteins that bind both HA and CSPGs. On the other surface, the C-terminus of the CSPG core protein directly interacts with trimeric TNR, creating the highly organized PNN structure [8, 9].

ACANs have an essential role in the formation of an intact and complex PNN structure, and ACAN-knockout animals show an abnormal PNN structure [10, 11]. HAPLN1 is a link protein and one of the key elements of PNNs. PNN formation is triggered by the expression of HAPLN1 and PNNs that lack HAPLN1 do not form properly [8, 12].

TNR is co-localized with PNNs, and provides a link between HA and glycoproteins [13–16]. TNR can promote or inhibit neurite outgrowth and neural and glial adhesion, depending on both the cell type and the other proteins that it may interact with [17, 18]. Trimeric TNR protein can bind three lecticans at the same time, so this interaction strengthens the macromolecular meshwork of PNNs. TNR-deficient mice have abnormal PNNs, cognitive and motor function defects, and reduced axonal transduction [19, 20].

PNNs also have neuroprotective effects [7]. In neurodegenerative diseases such as Alzheimer’s disease, Parkinson’s disease, Huntington’s disease and amyotrophic lateral sclerosis (ALS), reduced expression of PNN components, disrupted PNN structure and neural function defects have been observed, along with a lesser than normal length and lower number of neurites [21, 22]. In these diseases, iron ions accumulate in the brain, triggering an increase of free radicals, which cause oxidative stress [23, 24]. PNNs have the capacity to bind a large amount of iron ions due to their polyanionic character, and can thus protect neurons by reducing oxidative stress [25]. It has also been shown that PNN-positive neurons are less affected by neurofibrillary degeneration, lipofuscin accumulation, and the toxicity caused by amyloid β accumulation in Alzheimer’s disease [26, 27].

In several neurodegenerative diseases, the expression of PNN elements is known to be downregulated, suggesting that PNNs may have a role in neural differentiation [28, 29]. Additionally, it is known that in many diseases characterized by neurite elongation defects, the integrity of PNNs is impaired and the expression levels of HAPLN1, TNR and ACAN (the main elements of PNNs) are reduced. Several extracellular matrix proteins have been shown to play a role in neuritogenesis regulation [8, 30], but it is not known whether the elements of PNNs affect neural differentiation. Based on this information, it is hypothesized that the constituents of PNNs may be related to the neurite elongation process.

In our study, we used the PC12 cell line derived from rat pheochromocytoma as a neural differentiation model. This line is commonly used in neurobiology because of the cells’ ability to represent the neurite elongation process. The neurites continue to elongate during the differentiation process, reaching their maximum length on day 7 [31]. We aimed to discover whether PNNs are associated with neural differentiation by determining the changes in the mRNA and protein levels of HAPLN1, TNR and ACAN throughout the neural differentiation process.

## Methods

### Cell culture

PC12 cells were proliferated in a complete medium containing high glucose Dulbecco’s modified Eagle medium (DMEM; Biochrom) with stable glutamine, 10% (*v*/v) horse serum (HS; Biochrom), 5% (v/v) heat-inactivated fetal calf serum (FCS; Biochrom), 1 mM sodium pyruvate, 100 U/ml penicillin and 0.1 mg/ml streptomycin. The cells were grown in 75-mm^2^ tissue culture flasks coated with poly-L-lysine hydrobromide (Sigma-Aldrich), and incubated at 37 °C in a 5% CO_2_ humidified atmosphere. The cell culture medium was changed every 2 days.

### Differentiation of the PC12 cell line

**Fig. 1:**
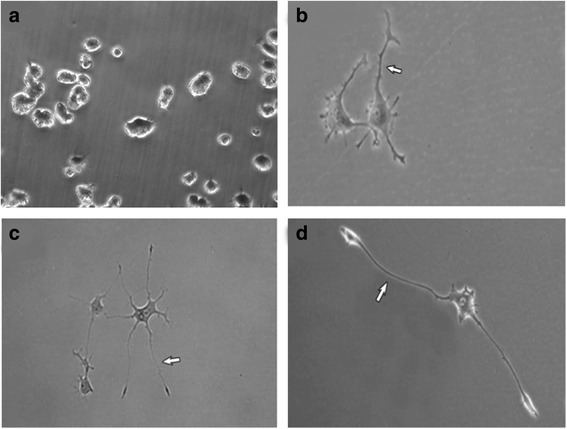
Images of PC12 cells before day 0 (**a**) and after differentiation for 3 days (**b**), 5 days (**c**) or 7 days (**d**). Arrows indicate the longest neurites (10×)

Cells were plated onto cell culture dishes (1 × 10^6^ cells/dish) for protein isolation, 6-well tissue culture plates (3 × 10^5^ cells/well) for RNA isolation, and 6-well plates (3 × 10^4^ cells/well) for immunofluorescent staining. The plates and dishes were coated with poly-L-lysine hydrobromide according to the manufacturer’s instructions. The cells were incubated for 3, 5 and 7 days (Fig. 1) in a differentiation medium containing high glucose DMEM with stable glutamine, 1% (*v*/v) HS, 1 mM sodium pyruvate, 100 U/ml penicillin, 0.1 mg/ml streptomycin and 50 ng/ml fresh nerve growth factor (NGF; Sigma-Aldrich).

### Quantitative real-time PCR

Cells were collected after differentiation (on day 3, 5 and 7) for RNA isolation, and maintained in RNAprotect Cell Reagent (Qiagen). RNA was extracted using the RNeasy Mini Kit (Qiagen), and the RNA concentration was determined with a NanoDrop Spectrophotometer (ThermoFisher Scientific). Reverse transcription was performed using QuantiTect Reverse Transcription Kit (Qiagen). The primers were:

- HAPLN1 forward: 5′ CTGGAGGATTATGGAAGATA 3′; and reverse: 5′ CACCACACCTTGTAACTCTA 3′
- TNR forward: 5′ CACTCCAAAGAACAATGAAG 3′; and reverse: 5′ GCTTGTTCCTTTAGTGCTAC 3′
- ACAN forward: 5′ GATACTCTCTGACATTTGAGG 3′; and reverse: 5′ GTATCTGACAGTCTGGTCCT 3′
- PPIA forward: 5′ TGTGCCAGGGTGGTGACTT 3′; and reverse: 5′ TCAAATTTCTCTCCGTAGATGGACTT 3′

The primers were used at a final concentration of 10 μM in each reaction. PCR was carried out using a Bio-Rad IQ5 Real-Time PCR System with SYBR Green Mix (Bio-Rad). Each sample was run in triplicate, using 96-well plates. The cycling conditions were: initial denaturation at 95 °C for 10 min, followed 40 cycles of 95 °C for 15 s (denaturation) and 60 °C for 1 min (annealing). HAPLN1, TNR and ACAN transcripts were normalized to PPIA, which is a commonly used housekeeping gene for PCR experiments [32].

### Western blot

Cells were scraped and lysed on days 3, 5 and 7 during differentiation in lysis buffer at pH 7.4, consisting of 10 mM Trisma-base, 300 mM NaCl, 2 mM EDTA and 0.5% Triton-X-100 with 1 mini protease inhibitor tablet. Samples were then sonicated 10 times at 50% amplitude for 20 s and centrifuged at 17000 g for 3 min at 4 °C. Protein concentration was determined using the bicinchoninic acid assay.

Proteins were denatured with 2× Laemmli buffer and boiled at 100 °C for 5 min. The samples were run on 10% sodium dodecyl sulfate polyacrylamide (SDS-PAGE) gel and blotted onto nitrocellulose membrane (ThermoFisher Scientific). The membranes were blocked with 10% non-fat dry milk in Tris-buffered saline with 0.2% Tween-20 (0.2% TBS-T) for 1 h. The following antibodies were used: anti-HAPLN1 (1:1000; Novus Biologicals NBP1–59150), rabbit polyclonal anti-ACAN (1:500; Merck AB1031), mouse monoclonal anti-TNR (clone #619 MAB1624, 1:500; R&D Systems), and mouse monoclonal anti-GAPDH (1:5000; Sigma). The blots were incubated in primary antibody overnight at 4 °C, and then incubated with horseradish peroxidase-coupled secondary antibodies (Invitrogen) at a dilution of 1:1000 for 1 h at room temperature (RT; 23 °C). They were then rinsed three times in 0.2% TBS-T, and the signals were visualized using the SuperSignal West Femto Maximum Sensitivity Substrate (ThermoFisher Scientific).

### Immunofluorescent staining

Cells were fixed for 20 min with 4% paraformaldehyde in 1× phosphate buffered saline (PBS) on days 3, 5 and 7, and permeabilized in 0.2% Triton-X-100 containing 1× PBS. After blocking with 10% bovine serum albumin (BSA) for 1 h, the cells were incubated overnight at 4 °C in blocking solution containing the following primary antibodies: anti-HAPLN1 (1:250; Novus Biologicals NBP1–59150), rabbit polyclonal anti-ACAN (1:100; Chemicon AB1031), and mouse monoclonal anti-TNR (clone #619 MAB1624, 1:50; R&D Systems). The cells were then washed three times with 0.2% Tween-20 containing PBS-T, and incubated for 1 h at RT in a blocking solution containing secondary antibodies (1:1000; ThermoFisher Scientific). After incubation with secondary antibodies, the cells were washed in 1× PBS three times for 10 min, and incubated with 4′,6-diamidino-2-phenylindole (DAPI) to stain the nuclei for 1 min at RT. The coverslips were then imaged using a Carl-Zeiss Axioplan 2 fluorescent microscope.

### Data analysis

The changes in mRNA levels were analyzed using the Bio-Rad iQ5 2.1 Standard Edition Optical System Software*.* Undifferentiated cells (day 0) were used as a calibrator, and mean C_t_ values for the target genes were normalized against the PPIA housekeeping gene. The results obtained from the quantitative real-time PCR experiments were calculated using the 2^-ΔΔCt^ method. The intensity of the protein bands obtained from the western blot experiments was quantitated using ImageJ (1.48 version). The results were compared to undifferentiated cells and glyceraldehyde 3-phosphate dehydrogenase (GAPDH) was used as a loading control.

The changes in gene expression and protein amounts were statistically analyzed with the non-parametric Kruskal-Wallis test, using the GraphPad Prism (6.01) software. Values *p* < 0.05 were considered significant. The time points of differentiation (days 3, 5, 7) were compared to the undifferentiated state (day 0) with Dunn’s multiple comparison test*.* Graphics were drawn with mean values using the GraphPad Prism (6.01) software with error bars equivalent to the standard error of the mean (SEM).

## Results

Our aim was to investigate the mRNA and protein levels of HAPLN1, TNR and ACAN in neural differentiation using PC12 cells.

**Fig. 2:**
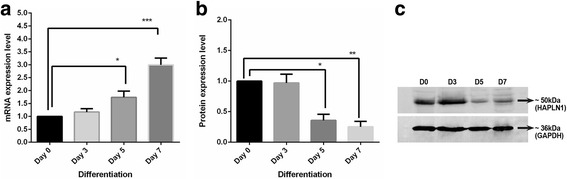
The expression analysis of HAPLN1 during differentiation at the mRNA level (**a**) and protein level (**b**). Representative western blot analysis of HAPLN1 protein (**c**). mRNA levels were normalized against the PPIA gene. GAPDH was used as a loading control in western blot experiments. *p < 0.05, **p < 0.01, ***p < 0.001

On days 5 and 7, 1.7-fold (*p* < 0.05) and 2.9-fold (*p* < 0.0001) increases in HAPLN1 mRNA levels were respectively observed compared to the non-differentiated state (Fig. 2a). For day 3, no significant change in the expression level was observed (1.2-fold). In contrast to the mRNA results, the intracellular HAPLN1 levels significantly decreased on days 5 (0.3-fold, *p* = 0.0017) and 7 (0.2-fold, p = 0.0017; Fig. 2b and c).

**Fig. 3:**
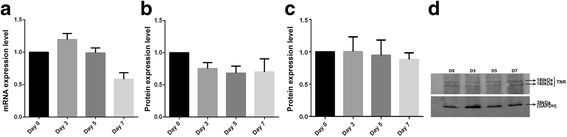
The expression analysis of TNR during differentiation at the mRNA level (**a**) and the protein levels for TNR-160 kDa (**b**) and TNR-180 kDa (**c**). Representative western blot analysis of TNR protein (**d**). mRNA levels were normalized against the PPIA gene. GAPDH was used as a loading control in western blot experiments

During differentiation, the expression levels of TNR remained unchanged (Fig. 3a). Consistent with the mRNA data, levels of both alternative splicing isoforms of TNR (160 and 180 kDa) did not change before or after differentiation (Fig. 3b–d).

**Fig. 4:**
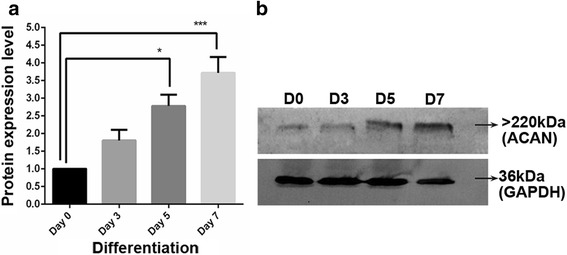
The expression analysis of ACAN during differentiation at the protein level (**a**). Representative western blot analysis of ACAN protein (**b**). GAPDH was used as a loading control in western blot experiments. *p < 0.05, **p < 0.01, ***p < 0.001

ACAN levels could not be determined using quantitative real-time PCR during the differentiation period. Interestingly, although the mRNA level was not detectable, western blot results show an increase in the intracellular ACAN level: 2.7-fold on day 5 (*p* < 0.05) and 3.7-fold on day 7 (*p* < 0.001; Fig. 4a and b).

**Fig. 5:**
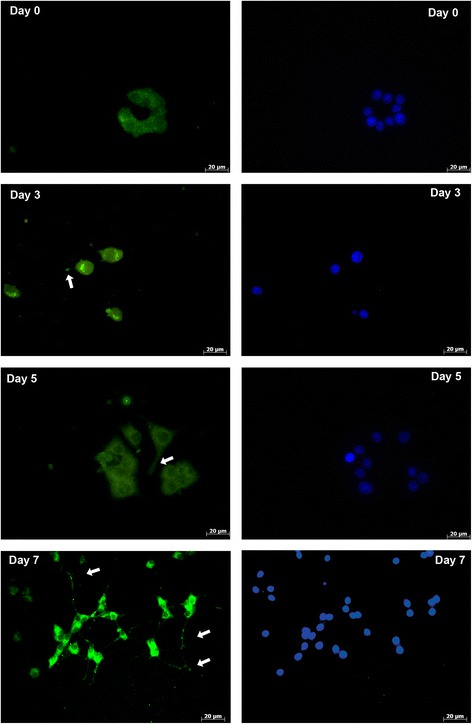
Immunofluorescent analysis of HAPLN1 before differentiation (day 0) and after differentiation for 3 days, 5 days or 7 days. Green/FITC: HAPLN1; Blue: DAPI for nuclei staining. Arrows indicates the neurites. Scale bar: 20 μm

**Fig. 6:**
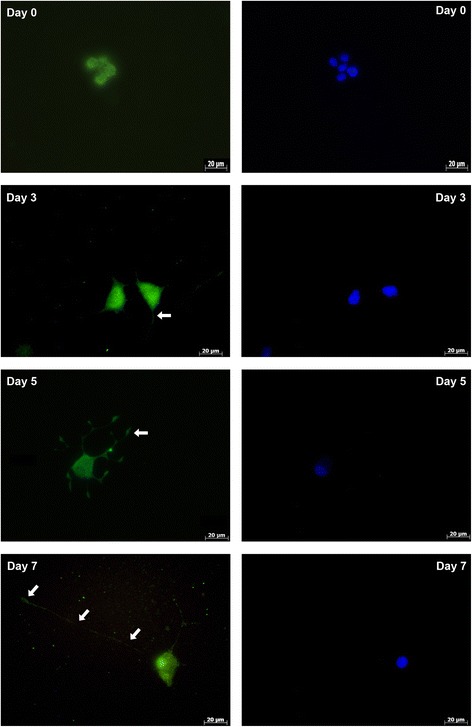
Immunofluorescent analysis of ACAN before differentiation (day 0), and after differentiation for 3 days, 5 days or 7 days. Green/FITC: ACAN; Blue: DAPI for nuclei staining. Arrows indicates the neurites. Scale bar: 20 μm

**Fig. 7:**
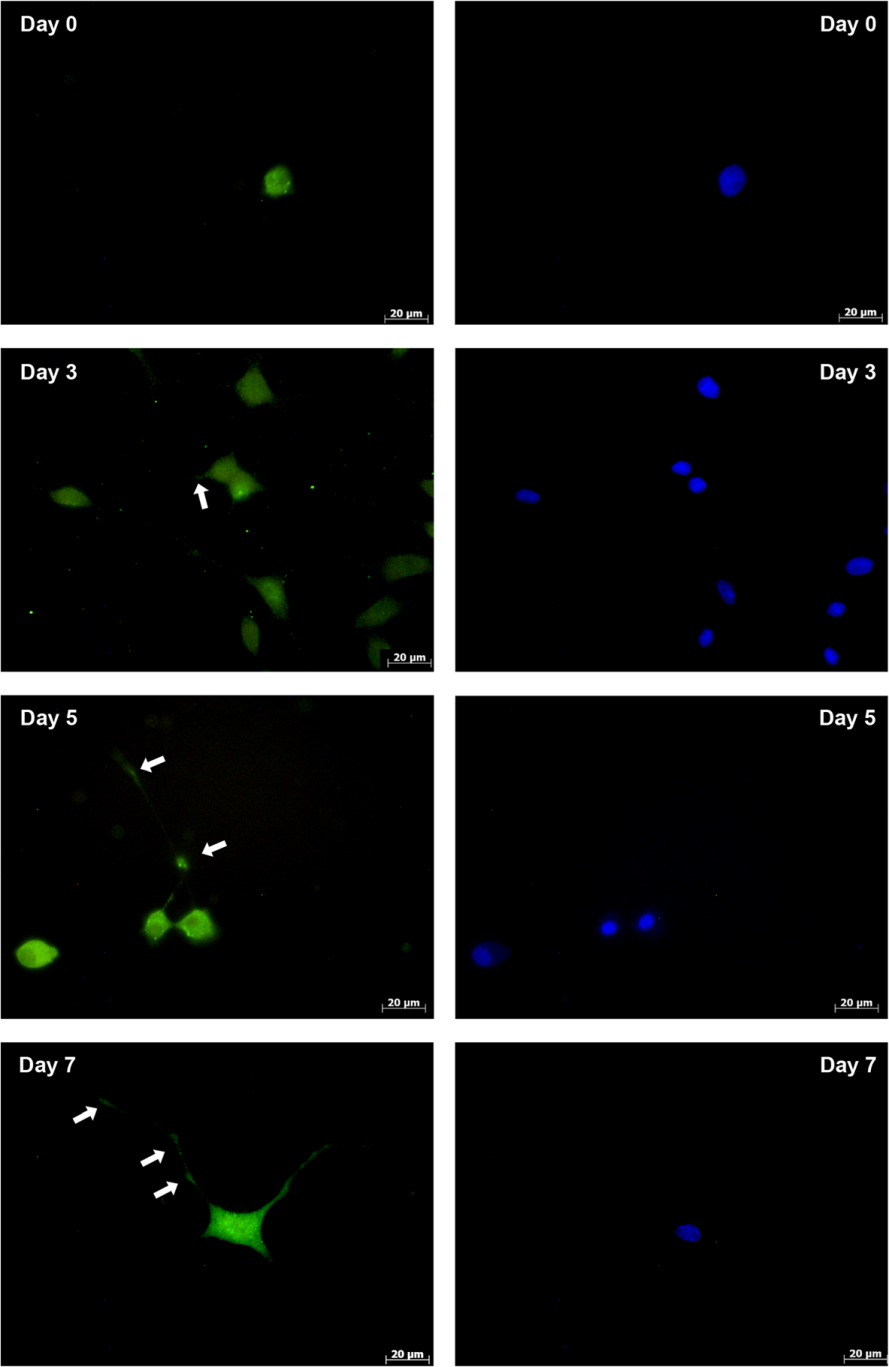
Immunofluorescent analysis of TNR before differentiation (day 0), and after differentiation for 3 days, 5 days or 7 days. Green/FITC: TNR; Blue: DAPI for nuclei staining. Arrows indicates the neurites. Scale bar: 20 μm

Immunofluorescent staining of HAPLN1 and ACAN showed that while the proteins were localized in the cytoplasm in an undifferentiated state, they spread out along the neurites during differentiation. They showed localization in both the cytoplasm and neurites in differentiated cells (Figs. 5 and 6). TNR was shown to localize mainly in the cytoplasm, but a less immunofluorescent signal was obtained at the neurite tips in differentiated cells as well (Fig. 7).

## Discussion

It has been shown that extracellular space contributes to the pathophysiology of diseases affecting the central nervous system (CNS), but the exact role of this extracellular matrix in such pathologies remains unclear.

Formation of PNNs begins in the embryonic period and progresses throughout the developmental period. The expression and secretion of the elements and their participation in the organization and structure of the PNNs occur at different time points [33]. Moreover, the participation of these elements differs under in vivo and in vitro conditions [5]. The compact structure of PNNs with all components in place does not form until adulthood [34].

Many studies have shown that the PC12 cell line is a widely accepted model for neural differentiation [35–38] and various neurodegenerative diseases. The capacity of neural differentiation to sympathetic and dopaminergic neurons in response to nerve growth factor is a useful and important feature of PC12 cells [39]. PC12 cells have also been used in neuroprotection studies [40]. In this study, we investigated the expression of PNN components at the RNA and protein levels during neural differentiation using the PC12 cell line as an in vitro model.

HAPLN1, which is one of the major PNN elements, interacts with both hyaluronan (HA) and CSPGs and stabilizes the interaction between the HA backbone and proteoglycans to subsequently allow the participation of other elements. It is known that HAPLN1 expression triggers the formation of PNNs, and in the absence of HAPLN1, PNN formation cannot occur correctly [5, 8]. Carulli et al. [33] showed that HAPLN1 initiates PNN nucleation as soon as it is secreted.

In our study, we determined that HAPLN1 expression increases during differentiation, whereas the intracellular protein level decreases. This suggests that after differentiation, HAPLN1 is secreted to the extracellular space as soon as it is synthesized. It was previously determined that HAPLN1 has a role in the initiation of the formation of PNNs, and in triggering nucleation [1, 41]. Our results are consistent with this report. Immunofluorescent analysis, which also supports these findings, showed that during differentiation, HAPLN1 is localized to the intracellular area on day 3, while it appears to be closer to the membrane on days 5 and 7, in line with the time point for its secretion.

TNR has a HA-binding domain in the N-terminus and a glycoprotein-binding domain in the C-terminus. Therefore, interaction between HA and glycoprotein in the PNN structure can be mediated by TNR [13] through its two active domains. While its FNIII domain supports adhesion and neurite outgrowth, its EGF domain inhibits neuritogenesis. However, the function of the whole protein is still unknown [42].

In our study, we did not determine any significant changes in the TNR mRNA and protein levels during the 7 days of differentiation. This may indicate that TNR has no role in neural differentiation. Giamanco et al. [9] showed that TNR participates in PNNs at a later time point than other components. Thus, the 7-day period may not be enough to determine whether TNR has a role in the differentiation process.

ACAN, a member of the lectican family, is a major element of PNNs, and is the most abundant protein in the CNS. The ACAN protein mainly consists of glycosaminoglycan (GAG) chains, such as chondroitin sulfate (CS) chains, N- and O- oligosaccharides, and keratan sulfate (KS) chains [13, 43]. It has a key role in neuroprotection and the restriction of synaptic plasticity. Giamanco et al. [4] reported that ACAN-knockout mice die after birth.

Despite the low levels of ACAN mRNA, we determined a significant increase in protein levels, suggesting that ACAN may be necessary for proper differentiation. Since ACAN has several post-translational modifications [10, 44], more time might be required before its secretion, and ACAN protein’s stability might be increased due to these modifications. Furthermore, it is known that the rate of mRNA production in mammalian cells is much lower than the protein production rate [45], and the regulation of transcripts at the genetic level takes much longer than at the level of proteins [46, 47]. In addition, ACAN protein may be a long-lived protein that gets accumulated over time while its mRNA becomes degraded. These interpretations may explain why there is an increase in the protein level even though the mRNA is synthesized at a baseline level.

It has been shown that in many studies, the protein and mRNA quantities do not always correlate, as their levels can vary from thousands to hundreds of millions of copies per cell [45, 48, 49]. The RNA-to-protein ratio may alter between different genes because an mRNA copy of a given gene can generate thousands of protein copies, while an mRNA copy of another gene generates relatively fewer protein copies [50].

## Conclusions

In our study, using PC12 cells as an in vitro model, we analyzed whether the expression of main components of PNNs are altered during neural differentiation. To confirm our preliminary results, further investigations will be performed to elucidate the role of these proteins in neurodegenerative disease models.

## Abbreviations

ACAN: aggrecan
ALS: Amyotrophic lateral sclerosis
BSA: Bovine serum albumin
CSPGs: Chondroitin sulfate proteoglycans
DMEM: Dulbecco’s modified Eagle medium
ECM: Extracellular matrix
FCS: Fetal calf serum
HA: Hyaluronan
HAPLN1: Hyaluronan and proteoglycan link protein 1
HS: Horse serum
HSPGs: Heparan sulfate proteoglycans
NGF: Nerve growth factor
PNNs: Perineuronal nets
TNR: Tenascin-R
